# Implicit assumptions and interpretation bias in youth with severe, chronic social phobia

**DOI:** 10.1007/s00787-021-01879-3

**Published:** 2021-10-01

**Authors:** Lisa Krömer, Tomasz A. Jarczok, Heike Althen, Andreas M. Mühlherr, Vanessa Howland, Stefanie M. Jungmann, Christine M. Freitag

**Affiliations:** 1 Department of Child and Adolescent Psychiatry, Psychosomatics and Psychotherapy, University Hospital Frankfurt, Goethe University, 60528 Frankfurt am Main, Germany; 2 Department of Neonatology and Pediatric Intensive Care, University Medicine Greifswald, 17475 Greifswald, Germany; 3 Department of Clinical Psychology, Psychotherapy and Experimental Psychopathology, Johannes Gutenberg-University Mainz, 55122 Mainz, Germany

**Keywords:** Affective Misattribution Procedure, Dysfunctional social assumptions, Implicit Association Test, Implicit Processing, Interpretation Bias, Social Phobia

## Abstract

Interpretation bias and dysfunctional social assumptions are proposed to play a pivotal role in the development and maintenance of social phobia (SP), especially in youth. In this study, we aimed to investigate disorder-specific implicit assumptions of rejection and implicit interpretation bias in youth with severe, chronic SP and healthy controls (CG). Twenty-seven youth with SP in inpatient/day-care treatment (*M* age = 15.6 years, 74% female) and 24 healthy controls (*M* age = 15.7 years, 54% female) were included. The Implicit Association Test (IAT) and the Affect Misattribution Procedure (AMP) were completed to assess implicit assumptions and interpretation bias related to the processing of social and affective stimuli. No group differences were observed for the IAT controlling for depressive symptoms in the analyses. However, group differences were found regarding interpretation bias (*p* = .017, *η*^*2*^_*p*_ = .137). Correlations between implicit scores and explicit questionnaire results were medium to large in the SP group (*r* =|.28| to |.54|, *p*_all_ ≤ .05), but lower in the control group (*r* =|.04| to |.46|, *p*_all_ ≤ .05). Our results confirm the finding of an interpretation bias in youth SP, especially regarding the implicit processing of faces, whereas implicit dysfunctional social assumptions of being rejected do not seem to be specific for SP. Future research should investigate the causal relationship of assumptions/interpretation bias and SP.

**Supplementary Information:**

The online version contains supplementary material available at 10.1007/s00787-021-01879-3.

## Introduction

Anxiety disorders are the most common mental disorders in children and youth [1]. In particular, social phobia (SP) frequently occurs in youth with a prevalence of up to 10% [2–5]. According to the International Classification of Diseases and Related Health Problems (10th edition, ICD-10 [6]), SP describes the fear of scrutiny by other people leading to the avoidance of social situations and is associated with the fear of criticism and low self-esteem. SP has a strong impact on social integration, schooling and vocational training and often persists into adulthood if left untreated [7–9].

Youth is a crucial phase for brain development and many cognitive and social changes occur [10]. The reliance on peers is getting more important, leading to increased autonomy from the family of origin, but also coming along with enhanced vulnerability for the development of social anxiety [10]. The importance of this developmental phase is underlined by a peak of social phobia onset in early youth [10]. Several aetiological models have been put forward to explain the occurrence and maintenance of SP [10]. The Cognitive Model of Social Anxiety Disorder has been used as basis for SP specific behavioural intervention approaches [11]. The model assumes, that in an individual with SP, a social situation will trigger assumptions and beliefs about the individual’s performance and behaviour. If these assumptions are negative, this will lead to the perception of the respective social situation as being dangerous, resulting in negative automatic thoughts and concerns that one’s standards of performance may not be achieved [11]. As a result, negative expectations towards further social situations are accumulating, leading to even more negative assumptions and cognitive biases, such as an interpretation bias [10]*.* The Cognitive Model of Social Anxiety Disorder has also been suggested to be applicable to youth, taking possible youth-specific factors into account, such as the role of social media, parental factors and peer victimisation [10].

Our study focusses on two crucial elements of the maintenance of SP: negative cognitive assumptions and interpretation bias. In patients with SP, negative assumptions are likely to evolve from firm beliefs about social integration and behaviour, which often include social rejection [10]. Against this background, social situations trigger biased interpretations, e.g. facial expressions are more likely to be interpreted as displaying negative instead of neutral or positive emotions [12]. In line with the Cognitive Model of Social Anxiety Disorders [11], negative cognitive assumptions and interpretation bias are assumed to be associated positively. To date, only a few studies have examined cognitive assumptions in SP. In adults, more negative social assumptions have been shown for highly socially anxious individuals than for low socially anxious participants [13]. In youths with social anxiety, similar results have also been found; one study in the general population [14] found that highly socially anxious youth, aged 14–20 years, showed more dysfunctional social assumptions than low socially anxious youth. Dysfunctional social assumptions have also been shown to predict the severity of social anxiety beyond depression and sociodemographic variables [14]. These negative assumptions about oneself can lead to the interpretation of ambiguous social cues as threatening and socially rejecting [15]. The “tendency to assign a threatening meaning to an objectively ambiguous stimulus with several possible interpretations” is called interpretation bias [16]. The interpretation bias has been studied widely in adults with SP [17–19], but only a few studies have included youth. Highly socially anxious adults have been consistently shown to interpret ambiguous social situations (e.g. receiving no answer to a birthday invitation) more negatively than less socially anxious adults [20, 21]; in youth, similar results have also been found [22]. For example, highly socially anxious youth aged 12–16 years, compared to non-socially anxious individuals, interpreted ambiguous social situations (e.g. no definite confirmation of an invitation) more negatively [22]; in another study, a similar group aged 12–17 years, interpreted ambiguous social scenes with a photograph of themselves inserted as the protagonist more negatively than low socially anxious individuals [23]. For adults, a misinterpretation was found not only for neutral–ambiguous stimuli, but also for negative social stimuli. One study found that young adults with higher social anxiety interpreted subtle negative emotional facial expressions more strongly in terms of a negative emotion and as threatening than adults with low social anxiety [24]. In addition, for positive social events, it was found that socially anxious adults endorsed more negative interpretations than patients with other anxiety disorders [25]. When taking a closer look at the interpretation of facial expressions in adults, only a few studies exist which present heterogeneous results. On the one hand, one study did not find differences in the interpretation accuracy of facial expressions [26], while, on the other hand, socially anxious adults were found to be more accurate at identifying facial expressions than healthy controls [27]. Furthermore, socially anxious adults were found to misinterpret expressions, e.g. misinterpreting faces showing disgust as expressing contempt [28], an emotion that is closely related to experiencing rejection [29]. A study on socially anxious children (aged 8–12 years) found no tendency to interpret neutral or positive facial expressions more negatively than healthy controls [12]. Since youth is the age-of-onset for SP, it should be of increasing interest to take a closer look at the interpretation of facial expressions during this crucial developmental phase.

Previous studies on dysfunctional assumptions and negative interpretation bias in SP were mostly based on explicit self-report measures or questionnaires [22]. Dual-processing models, however, underline the importance of two information processing modes: the explicit and the implicit information processing mode [30–32]. The explicit mode is described as a reflective, conscious or effortful information processing, whereas the implicit mode is defined as a more automatic, quick or unconscious processing [30, 33]. Implicit processing was shown to be of importance in many forms of psychopathology [34] and is likely to play a particularly relevant role in youth SP [35]. Youth with SP experience a high level of self-presentational concerns and fear of others' evaluations; this may cause inaccurate or incomplete answers on self-reports [36]. Implicit measures are less susceptible to response biases (e.g. social desirability or response tendencies to, for example, medium responses) due to concealing the intention of the measurement and they also do not depend on introspective abilities [33]. These measures are based on behavioural data (e.g. reaction times of categorisations as in the Implicit Association Test, IAT [37]) or on ratings that do not relate to the characteristic of interest (e.g. Chinese characters as in the Affect Misattribution Procedure, AMP [38]). The IAT is proposed to assess associations between a category (e.g. self) and attribute (e.g. rejection), from which conclusions on a person’s assumptions, beliefs or schemas can be drawn [39]. The IAT was shown to have the best validity and reliability of seven different implicit self-attribution measures [40]. The AMP assesses automatically activated responses based on a misattribution a person makes about the source of its affect or cognition [41], relying on the fact that people have difficulties disentangling their affective responses when two events occur shortly after each other [42]. The AMP has been used to assess implicit biases in earlier studies on adults with psychiatric disorders [43, 44]. For the AMP, construct validity and predictive validity with explicit attitude measures were shown [42]. Since implicit assumptions and automatic retrieved implicit biases are proposed to play a relevant role in SP and as both measures have been studied in the context of SP [45, 46], IAT and AMP seem to be the most suitable measuring instruments for the present study’s endeavours.

With regard to implicit biases, it has been shown that patients with hypochondriasis show significantly more negative affective reactions in illness prime trials than controls, concluding that negative affective evaluation bias is a unique feature of hypochondriasis [43]. In addition, implicit bias has been studied in patients with eating disorders; these patients showed a higher negative implicit bias for high-calorie food than the healthy controls [44].

Concerning assumptions in SP, the assumptions of being rejected may be of particular importance for SP. Interpersonal rejection sensitivity was found to be not only a specific aspect of depression, but also of SP [47]. In adults with social anxiety disorder, higher rates of explicit interpersonal rejection sensitivity were found compared to healthy controls, independent of self-reported depression [47]. Regarding the implicit assumption of being rejected, only one single study in adults compared SP with healthy controls. Adults with SP and comorbid depression implicitly considered themselves as more socially rejected compared to the SP and control groups; however, the latter two groups did not differ [46]. To our knowledge, no studies exist concerning implicit assumptions of being rejected in youth. A study in youth outpatients with SP (aged 14–20 years) which used the AMP [38] and the IAT (e.g. self-referent words as prime stimuli for assessing implicit self-esteem) [45] to focus on implicit self-esteem did not observe any group differences to the controls. Since explicit assumptions of rejection were found to play a role in SP and implicit assumptions have so rarely been studied, it seems to be of particular importance to examine this further, especially in the vulnerable developmental phase of youth.

Thus, the aim of the current study was to investigate dysfunctional implicit social assumptions and implicit interpretation bias regarding social and emotional stimuli in youth with severe SP in inpatient/day-care hospital treatment. In the present study, a specific version of the AMP to study interpretation bias in reaction to emotional facial expression and a version of the IAT testing implicit assumptions regarding social rejection were implemented. We first hypothesised that youth with SP show stronger implicit assumptions about oneself of being socially rejected than healthy controls. In addition, these implicit assumptions of being rejected are hypothesised to be positively correlated with the explicitly assessed severity of anxiety and SP symptoms. In line with the findings on explicit interpretation bias in adults with SP, we hypothesised that youth with SP, compared to healthy controls, show a stronger implicit interpretation bias, resulting in a more negative interpretation of emotional faces. The resulting pleasantness, experienced when confronted with the facial expressions, is hypothesised to be negatively correlated with the explicitly assessed severity of anxiety and SP symptoms.

## Methods

### Participants

The participants of the SP group (SPG) were recruited at the Department of Child and Adolescent Psychiatry, Psychosomatics and Psychotherapy, University Hospital Frankfurt, Goethe University, while the participants of the control group (CG) were recruited from the general population via social media and advertisements. All participants were aged between 12 and 17 years. Exclusion criteria were an IQ < 70, uncorrected vision (computer-based tasks) and language proficiency in Chinese/Mandarin (due to the AMP using Chinese characters). IQ was assessed by calculating the mean score of the Vocabulary and Matrix Reasoning subtests of the Wechsler Intelligence Test for Children (WISC-IV) [48] or the Wechsler Intelligence Test for Adults (WAIS-IV; > 16; 11 years) for each participant [49]. Additional exclusion criteria of the SPG were a diagnosis of obsessive–compulsive disorder, tic disorder, acute post-traumatic stress disorder, conduct disorder, bipolar disorder, autism spectrum disorder, substance use disorder, psychotic symptoms and disorders and suicidal ideation. The CG was assessed for the absence of psychiatric symptoms by the Youth Self Report [50] and the Child Behavior Checklist [51]. Controls were excluded when at least one subscale of one of the questionnaires indicated clinically relevant symptoms.

The SPG included 27 youth (*M* age = 15.58 years, SD = 1.29 years, 74% female) with a diagnosis of social phobia according to the ICD-10 [6]. The youth were seeking inpatient or day-care hospital treatment due to school absenteeism in connection with their psychopathology. Diagnoses and information on school absenteeism were provided by experienced child and adolescent psychiatrists or clinical child psychologists, based on the information from direct interviews with the patient or care givers, information by the multi-professional team and behaviour observation. Comorbid diagnoses according to ICD-10 [8] were depressive episodes or recurrent depressive disorder (*n* = 24), other anxiety disorders (*n* = 7), eating disorders (*n* = 2), somatoform disorder (*n* = 2), developmental disorder of speech (*n* = 3), reading disorder (*n* = 1) and attention-deficit hyperactivity disorder (*n* = 1). Six of the patients received antidepressant medication (Sertralin, Citalopram, Escitalopram) while taking part in our study. The CG included 24 typically developing youth (*M* age = 15.68 years, SD = 2.04 years, 54% female).

### Procedure

The study was approved by the local ethical committee of the Medical Faculty of the Goethe University Frankfurt am Main. Written informed consent was obtained from both parents and youth. First, participants received paper–pencil questionnaires once consent had been obtained and returned the completed questionnaires. Subsequently, the participants took part in an experimental session and completed the IAT [37] and the AMP [38]. To conduct a manipulation check of the stimuli used in the AMP [38], 35 of the participants (12 patients, 17 controls) completed an explicit rating of the face stimuli using the Self-Assessment Manikin Rating (SAM) afterwards [52, 53].

### Measures—Experimental tasks

The *Implicit Association Test* (IAT [32]) is a computer-based reaction time paradigm measuring implicit associations between target and attribute categories. In this study, we used the IAT for implicit social assumptions (IAT-SP), similar to the study by Wong and colleagues [46] in adults. The IAT-SP investigates the target categories of *self* (I, own, my, me, self) and *other* (them, others, you, your, they) and the attribute categories of *rejection* (forgotten, alienated, deserted, shunned, disliked) and *acceptance* (loved, welcomed, admired, included, respected). The participants were instructed to assign a word belonging to either a target category or an attribute category (e.g. “me” as representing target category “self”) presented in the middle of the screen to the correct one of the opposing categories presented in the left and right upper corners of the screen (e.g. left: “self” vs. right: “other”) as fast as possible. The participants used keys on the keyboard to assign the words to the categories with left-sided keys corresponding to the category in the left corner and right-sided keys to the right corner (e.g. “me” =  > “self” in left corner =  > left button press). When the answer was incorrect a red “X” appeared and they were asked to assign it to the correct category. Within the IAT-SP there were five blocks of trials. The first, second and fourth blocks were practise blocks, where one category was presented in each upper corner (e.g. left: “self” vs. right: “other”). Each practise block consisted of 24 trials. The third and fifth blocks were critical test blocks, where category combinations (e.g. “self + acceptance” in upper left corner; “other + rejection” in upper right corner) were presented and the word displayed in the middle of the screen had to be assigned to one of the category combinations (e.g. “me” =  > “self + acceptance”). The sides of the presented category combinations were switched between the third and fifth blocks. Each test block consisted of 24 practise trials and 40 test trials. Four different versions of the IAT-SP existed to counterbalance all possible combinations of the categories (e.g. “self”, “other” and “acceptance”, “rejection”) and sides (right or left upper corner). The versions are presented in the supplement (Online Resource 1). The internal consistencies of the reaction times of the IAT-SP in our study ranged from Cronbach’s *α* = 0.83 to *α* = 0.94.

For the IAT, the D_1_ score as an IAT effect was computed according to the published algorithm [54]. All response latencies > 10.000 ms and < 300 ms were first deleted and response latencies for errors were included in the calculation. All response latencies of practise and test trials of the critical blocks (blocks 3 and 5) were used. The mean latencies for the critical blocks for each IAT and the individual-respondent standard deviations were computed first. In the next step, the mean response latencies for the “Self/Rejection” associations were subtracted from the “Other/Rejection” mean response latencies. This difference was then divided by the pooled standard deviation to obtain the D_1_ score. Higher values in the IAT D_1_ score, thus, correspond to a higher implicit assumption of feeling socially rejected.

The *Affect Misattribution Procedure* (AMP [33]) is an implicit computer-based paradigm that combines the procedure of computer-based priming experiments and the logic of projective tests. The AMP taken in this study was adapted for social stimuli [38]. In the AMP [38], primes (pictures of faces) are presented supraliminally to the participants and, subsequently, an ambiguous target stimulus (Chinese character) is shown; the prime was presented for 33 ms, followed by a blank screen for 466 ms, the ambiguous stimulus for 117 ms and a mask sign (black and white pattern) during which the participants had to answer. All stimuli were presented in the centre of the screen. The participants were told to rate the ambiguous stimulus on a 4-point Likert scale ranging from *very unpleasant* (1) to *very pleasant* (4) and were directed to ignore the prime. The primes used were pictures of young adults showing angry, fearful, happy or neutral emotional facial expressions (taken from the Radboud Faces Database) [55]. The AMP [38] consisted of 38 trials for each of the four emotional facial expressions [4 emotions (angry, fearful, happy, neutral) × 38 = 152 trials], half male and half female pictures. Across six previous studies, the AMP showed a mean Cronbach’s *α* = 0.88 [38]. In our study, the internal consistencies for the AMP ranged from *α* = 0.77 to *α* = 0.95. For the AMP, we assessed the pleasantness of the Chinese characters for each emotional face category (angry, fear, happy, neutral) by computing the pleasantness mean score for each emotion separately and additionally computed a pleasantness total score by calculating the mean of the pleasantness responses for all four emotions for each participant.

In addition, explicit ratings (valence and arousal) of the emotional faces were assessed to examine the validity of the prime stimuli used in the AMP. A subsample (*N* patients = 14, *N* controls = 21) rated the valence and arousal of all the primes. The emotional facial expressions were presented to the participants in the middle of the screen and the participants were asked to rate the prime first on valence (nine pictures ranging from sad to happy, with a scale of 1–9 underneath) and, second, on arousal (nine pictures ranging from calm to excited, with a scale of 1–9 underneath) using the non-verbal Self-Assessment Manikin (SAM [52]).

### Measures—Questionnaires

The *Diagnostic System for Mental Disorders* (DISYPS-II [56]) is a German rating system to assess the diagnosis and severity of different mental disorders in childhood and youth according to ICD-10 and DSM-5. For our study, the total score of the 33-item self-report questionnaire for *anxiety disorders (SBB-ANZ)* and the social anxiety subscale score were used to measure the severity of explicit general anxiety and social anxiety. All items were answered on a four-point Likert scale from 0 = “not at all” to 3 = “particularly true”. The total scale showed good convergent and discriminant validity and acceptable to high internal consistency (*α* = 0.59–0.92) [56, 57]. In our study, internal consistencies for the SBB-ANZ total score were Cronbach’s *α* = 0.85 for the SPG and Cronbach’s *α* = 0.79 for the CG.

The German version of the *Social Phobia and Anxiety Inventory for Children* (SPAI-C [58]) is a 26-item self-report assessing the severity of social anxiety in children and youth. Somatic, cognitive and behavioural aspects of social anxiety are rated on a 3-point Likert-scale ranging from “never” to “mostly/always”. Very good internal consistency (*α* = 0.92—0.95) and convergent and discriminant validity could be shown for clinical and non-clinical samples [59, 60]. In our study, internal consistencies of *α* = 0.95 for the SPG and *α* = 0.92 for the controls were obtained.

The *Beck’s Depression Inventory* (BDI-II [61]) is a self-report measure assessing the severity of depressive symptoms. The 21-item questionnaire is based on DSM-IV criteria and showed very good internal consistencies (*α* = 0.84–0.94) in clinical and non-clinical samples and sufficient retest–reliability (*α* > 0.75) [62]. In addition, the BDI-II shows high convergent and content validity [62, 63]. In our study, the internal consistencies of the BDI-II for patients were *α* = 0.93 and for controls *α* = 0.72.

The following questionnaires were obtained from the typically developing controls to check the exclusion criteria.

The German version of the *Youth Self Report* (YSR 11–18 [50]) is a self-report questionnaire for youth aged 11–18 years. The items of the YSR 11–18 can be divided into eight problem scales (anxious/depressed, withdrawn/depressed, somatic complaints, social problems, thought problems, attention problems, rule-breaking behaviour and aggressive behaviour). In the present study, internal consistencies for the subscales ranged from *α* = 0.86 to *α* = 0.91.

The German version of the *Child Behavior Checklist* (CBCL 6–18 [51]) is a questionnaire for parents of children and youth aged 6–18 years with the same subscales as the YSR 11–18, assessing symptoms of the children from the parents’ point of view. In our study, the internal consistencies of the subscales ranged from *α* = 0.62 to *α* = 0.99.

### Statistics

All dependent variables were normally or approximately normally distributed. Descriptive data were compared by analysis of variance (ANOVA, i.e., age, IQ, BDI-II, SPAI-C and SBB-ANZ) or the Chi-square test (i.e., sex). To test the study's hypotheses, analyses of covariance (ANCOVA) with the D_1_ scores of the IAT-SP, the pleasantness total score of the AMP, or the explicit valence or explicit arousal, as dependent measures, were computed. Furthermore, we computed repeated measures ANCOVAs (rmANCOVA) with the AMP’s pleasantness score for each emotion separately, explicit valence ratings for each emotion separately and the explicit arousal ratings for each emotion separately, as dependent measures. The factor group was the predictor of interest, while the covariates were BDI-II and IQ in the rmANCOVAs. Due to some missing values of the covariates intelligence and depressive symptoms, the number of participants in the ANCOVA was slightly reduced compared to recruitment. For the AMP, initially, a model without the task related factor “emotions” was calculated, followed by a sensitivity analysis to test for group × emotions interaction. Bonferroni-adjusted post-hoc analyses were computed. Greenhouse–Geisser correction was used to correct for violations of sphericity and partial eta-square was calculated as effect size (*η*^2^_*p*_ ≥ 0.01 small effect; *η*^2^_*p*_ ≥ 0.06 medium effect; *η*^2^_*p*_ ≥ 0.14 large effect). *P* values < 0.05 were taken as criteria for rejection of H0. Due to the sample size which had a power of 80% to detect only a large effect size with an alpha < 0.05, no adjustment for multiple testing was done. To explore the role of depressive symptoms, the ANCOVAs of IAT-SP and AMP were repeated without BDI-II as the covariate. In addition, correlations between the implicit and explicit measures were assessed by computing partial correlations, controlling for the covariates BDI-II and IQ. Relevant correlations above |*r* ≥ 0.3| are discussed below.

## Results

### Participant characteristics

Demographic characteristics and questionnaire data of the SPG and the CG are presented in Table 1. The SPAI-C total score [*F*(1, 43) = 82.25, *p* < 0.001, *η*^*2*^_*p*_ = 0.66] and SBB-ANZ total score [*F*(1, 38) = 69.20, *p* < 0.001, *η*^*2*^_*p*_ = 0.65] were higher in the SPG than the CG. The groups did not differ with regard to sex **[***χ*^*2*^ (1) = 2.21; *p* = 0.138, *Φ* = 0.21] or age [*F*(1, 49) = 0.05, *p* = 0.832, *η*^*2*^_*p*_ = 0.01]*.* However, there were significant differences regarding IQ [*F*(1, 46) = 4.21, *p* = 0.046, *η*^*2*^_*p*_ = 0.08] and depressive symptoms (BDI-II) [*F*(1, 48) = 67.43, *p* < 0.001, *η*^*2*^_*p*_ = 0.58]. Therefore, IQ and BDI were used as covariates in further analyses.

**Table 1:** Sample description

Characteristics	SPG (*N* = 27)	CG (*N* = 24)	Statistics	
	*M* (SD*)*	*M* (SD)	*p*	Effect size
Age (years)	15.58 (1.29)	15.68 (2.04)	.832	*η*^2^_*p*_ = .01
Sex (% female)	74.1%	54.2%	.138	*Φ* = .21
IQ BDI-II (0–63) SPAI-C (0–52) SBB-ANZ (0–99)	102 (11.9) 27.22 (13.85) 31.34 (12.40) 10.75 (5.76)	109 (10.9) 3.46 (2.87) 4.23 (4.98) 0.52 (0.98)	.046 < .001 < .001 < .001	*η*^2^_*p*_ = .08 *η*^2^_*p*_ = .58 *η*^2^_*p*_ = .66 *η*^2^_*p*_ = .65

*SPG* Social Phobia group*; CG* Control group; *M* Mean score; *SD* Standard deviation; *N* Number of participants; *BDI-II* Beck’s Depression Inventory; *SPAI-C* Social Phobia and Anxiety Inventory for children/adolescents; *SBB-ANZ* total score of the self-report inventory for Anxiety Disorders; effect sizes (*η*^2^_*p*_ ≥ .01 small effect; *η*^2^_*p*_ ≥ .06 medium effect; *η*^2^_*p*_ ≥ .14 large effect)

### Experimental measures (IAT and AMP)

Results of the IAT and AMP by group are shown in Table 2. The ANCOVA for the IAT-SP showed that both groups did not differ regarding their implicit assumption of being rejected [*F*(1, 41) = 0.92, *p* = 0.343, *η*^*2*^_*p*_ = 0.022]. When the ANCOVA was repeated without BDI-II as the covariate, a difference with a medium effect size between SPG and CG was found [*F*(1, 43) = 2.94, *p* = 0.094, *η*^*2*^_*p*_ = 0.064]. This is reflected by the raw mean scores between groups (SPG D_1_-score: *M* = -0.12, *SD* = 0.45; CG D_1_-score: *M* = -0.29, *SD* = 0.23). This suggests greater assumptions of being rejected due to depressive symptoms, but not due to anxiety disorder, in patients with SP *and* depressive symptoms compared to healthy controls. Regarding the AMP, the ANCOVA showed differences between the pleasantness total scores of the groups [*F*(1, 39) = 6.19, *p* = 0.017, *η*^*2*^_*p*_ = 0.137]. The SPG showed lower pleasantness total scores (*M* = 2.31, SD = 0.32) than the CG (*M* = 2.64, SD = 0.43), which could be interpreted as a higher implicit interpretation bias in the SPG than in the CG. Repeating the ANCOVA without the factor BDI-II as the covariate did not change the results [*F*(1, 40) = 5.55, *p* = 0.024, *η*^*2*^_*p*_ = 0.122]. Adding the factor “emotion” to the rmANCOVA resulted in a significant main effect of emotion [*F*(1.6, 60.4) = 3.50, *p* = 0.048, *η*^*2*^_*p*_ = 0.082]. Pleasantness scores for the emotions were shown to be rated differently across all patients. However, no interaction of emotion and group [*F*(1.6, 60.4) = 0.39, *p* = 0.628, *η*^*2*^_*p*_ = 0.010] occurred and, again, group differences did not change [*F*(1, 39) = 6.19, *p* = 0.017, *η*^*2*^_*p*_ = 0.137] underlining the finding that a difference in the pleasantness score between the SPG and CG was independent of emotions.

**Table 2:** Cognitive test results and self-assessment by group—raw data

	SPG (*N* = 27)	CG (*N* = 24)
	*M* (SD*)*	*M* (SD)
IAT-SP D_1_ score	− .12 (.45)	− .29 (.23)
AMP pleasantness total score	2.31 (.32)	2.64 (.43)
AMP angry	2.11 (.47)	2.55(.46)
AMP fear	2.17 (.44)	2.61 (.47)
AMP happy	2.67 (.54)	2.76 (.43)
AMP neutral	2.30 (.39)	2.65 (.39)
SAM valence total score	4.76 (.51)	4.88 (.39)
SAM valence angry	3.70 (.95)	4.00 (.84)
SAM valence fear	3.76 (.77)	4.09 (.72)
SAM valence happy	7.22 (.99)	6.77 (.90)
SAM valence neutral	4.37 (.75)	4.65 (.45)
SAM arousal total score	5.41 (.83)	5.21 (1.47)
SAM arousal angry	6.37 (1.00)	4.58 (1.75)
SAM arousal fear	6.58 (1.13)	5.14 (1.94)
SAM arousal happy	4.43 (1.96)	3.84 (1.63)
SAM arousal neutral	4.27 (1.46)	3.27 (1.26)

*SPG* Social Phobia group; *CG* Control group; *M* Mean score; *SD* Standard deviation; *N *number of participants*; IAT-SP D*_*1*_* score* D_1_ score of the Implicit Association Test; *AMP angry, fear, happy, neutral* Pleasantness scores in the Affective Misattribution Procedure for each emotion (angry, fear, happy, neutral) separately, *SAM valence angry, fear, happy, neutral* Valence scores in the explicit rating using the Self-Assessment Manikin for each emotion separately (angry, fear, happy, neutral); *SAM arousal angry, fear, happy, neutral* Arousal scores in the explicit rating using the Self-Assessment Manikin for each emotion separately (angry, fear, happy, neutral)

### Explicit valence and arousal ratings (SAM) for facial expressions in the AMP

Results of the explicit valence and arousal ratings by group are shown in Table 2. Regarding the valence ratings, the ANCOVA could not demonstrate differences between the groups [*F*(1, 25) = 0.47, *p* = 0.498, *η*^*2*^_*p*_ = 0.019]. The rmANCOVAs showed no main effect of valence [*F*(1.7, 42.3) = 1.60, *p* = 0.216, *η*^*2*^_*p*_ = 0.06], no main effect of group [*F*(1, 25) = 0.47, *p* = 0.498, *η*^*2*^_*p*_ = 0.019] and no interaction effect of group and emotion [*F*(1.7, 42.3) = 0.17, *p* = 0.807, *η*^*2*^_*p*_ = 0.007]. The emotional faces were not rated differently on valence when participants were asked to rate them explicitly and the groups did not differ with regard to their valence rating. The results did not change when repeating the analyses without BDI-II as a covariate. With regard to arousal, the ANCOVA revealed no differences between the groups [*F*(1, 25) = 0.56, *p* = 0.463, *η*^*2*^_*p*_ = 0.022]. The rmANCOVA showed no main effect of arousal [*F*(2.1, 51.4) = 0.24, *p* = 0.798, *η*^*2*^_*p*_ = 0.009] and no interaction effect was found [*F*(2.1, 51.4) = 0.53, *p* = 0.599, *η*^*2*^_*p*_ = 0.021]. In addition, when explicitly rating the faces on arousal, no differences for the various emotional faces were obtained and no differences between the CG and SPG occurred. When repeating the analyses without BDI-II as a covariate, a group difference between the SPG and CG was found [*F*(1, 26) = 8.02, *p* = 0.009, *η*^*2*^_*p*_ = 0.236] suggesting a difference in the rating of arousal between youth with SP *and* depressive symptoms and healthy controls.

### Correlations of implicit and explicit social phobia measures

With regard to correlations of IAT-SP and explicit social phobia measures across all participants (SPG and CG), correlations above *|r* = 0.30| were observed between IAT-SP and SPAI-C (*r* = 0.530, *p* = 0.002; CI = [0.31; 0.87]), IAT-SP and the social phobia subscale of the SBB-ANZ (*r* = 0.53, *p* = 0.002; CI = [0.31; 0.87]) and IAT-SP and the SBB-ANZ total score (*r* = 0.364, *p* = 0.041, CI = [0.10; 0.66]). For the SPG, positive correlations above |r = 0.30| were shown between IAT-SP and SPAI-C (*r* = 0.54, *p* = 0.039; CI = [0.32; 0.88]), IAT-SP and the social phobia subscale of the SBB-ANZ (*r* = 0.51, *p* = 0.055; CI = [0.28; 0.84]) and IAT-SP and the SBB-ANZ total score (*r* = 0.35, *p* = 0.20, CI = [0.09; 0.65]). For the CG separately, correlations above |r = 0.3| were found between IAT-SP and SPAI-C (*r* = 0.46, *p* = 0.082; CI = [0.22; 0.78]), IAT-SP and the social phobia subscale of the SBB-ANZ (*r* = 0.44, *p* = 0.101; CI = [0.19; 0.75]) and IAT-SP and the SBB-ANZ total score (*r* = 0.45, *p* = 0.097, CI = [0.20; 0.76]). For AMP pleasantness, the total score and explicit social phobia measures across all participants revealed no correlations above |*r* = 0.3|, whereas for the SPG separately, correlations above |*r* = 0.3| were observed between the AMP pleasantness total score and SPAI-C total score (*r* = -0.50, *p* = 0.057, CI = [− 0.83; − 0.27]) and between AMP pleasantness total score and the SBB-ANZ total score (*r* = -0.39, *p* = 0.146, CI = [− 0.69; − 0.13]). For the CG separately, correlations above |*r* = 0.3| were shown for the AMP pleasantness total score and SBB-ANZ (*r* = 0.43, *p* = 0.113, CI = [0.18; 0.74]). All correlations are shown in Table 3.

**Table 3:** Partial correlations between implicit and explicit measures (self-reports)

	IAT-SP			AMP			SPAI-C			SBB-ANZ (SP subscale)			SBB-ANZ		
	SPG	CG	Total	SPG	CG	Total	SPG	CG	Total	SPG	CG	Total	SPG	CG	Total
1. IAT-SP	1	1	1												
2. AMP	− .17	− .17	− .27	1	1	1									
3. SPAI-C	.54*	.46	.53**	− .50	.10	− .27	1	1	1						
4. SBB-ANZ (SP subscale)	.51	.44	.53**	− .28	− .04	− .22	.76**	.41	.76***	1	1	1			
5. SBB-ANZ (total score)	.35	.45	.36*	− .39	.43	− .09	.70**	.31	.69***	.71**	.52*	.73***	1	1	1

*SP* Social Phobia; *SPG* Social Phobia group; *CG* Control group; *total* all participants; *IAT-SP* Implicit Association Test (social phobia version); *AMP total score* Affective Misattribution Procedure total pleasantness score; *SPAI-C* Social Phobia and Anxiety Inventory for children/adolescents; *SBB-ANZ SP subscale* score of the self-report inventory for Anxiety Disorders’ social phobia subscale; *SBB-ANZ* total score of the self-report inventory for Anxiety Disorders

*p < .05, **p < .01, ***p < .001

## Discussion

The aim of the study was to investigate implicit, disorder-specific, dysfunctional assumptions and interpretation bias in youth with severe, chronic SP. So far, these cognitive processes have not been investigated in such a severely affected group of youth and this study may contribute to closing this research gap. The scores in the self-reports confirmed the severity of the SP. Compared to other studies assessing youth with SP, our sample showed higher SPAI-C total scores [64, 65]. Furthermore, depressive symptoms were higher than in other comparable studies on youth with SP [45]. The controls showed similar mean SPAI-C and BDI-II scores compared to other studies with non-clinical samples [45, 64, 65].

We hypothesised that youth with severe, chronic SP showed stronger implicit assumptions about oneself of being rejected than healthy controls. The results of our study indicate no differences between the groups for the implicitly assessed assumptions of being rejected as measured by the IAT-SP. This result is in line with another study [46] which also found no differences between socially anxious adults and controls regarding implicit assumptions about being rejected. However, adults with SP and comorbid depression showed stronger implicit dysfunctional social assumptions compared to SP without comorbid depression and the controls [46]. When not controlling for depressive symptoms in our study, results similarly showed group differences with a medium effect size. It is worth mentioning that we examined a rather small sample, thus, the observed medium effect group difference observed when not controlling for depressive symptoms may have reached significance in a larger sample. Therefore, feelings of being rejected may be due to the frequently co-occurring depression in youth and adult SP. Rejection sensitivity has been discussed as a specific aspect for SP [47] and for depressive disorder [66, 67]. Given studies in depressed youth which replicated rejection sensitivity as a risk factor for depression [68–70], our results have to be interpreted as follows. We cannot confirm the hypothesis that the feeling of being rejected is specific to SP, but it likely plays a strong role in co-occurring depression in SP. The observed positive correlation of the explicit self-reported severity of SP (SPAI-C and SBB-ANZ social phobia subscale) with the implicit assumptions of being rejected (IAT-SP) were driven by the SPG. The highest correlations of the implicit assumptions of being rejected and explicit SP symptoms and anxiety, in general, were found in the SPG. The higher the level of SP symptoms and anxiety, the stronger the implicit assumption of social rejection seems to exist, but this connection is more likely to be present above a certain level of social phobia.

Second, we hypothesised that youth with SP compared to healthy controls show a stronger implicit interpretation bias, resulting in the more negative interpretation of emotional faces. In accordance with our hypothesis, the SP group showed lower pleasantness responses than the control group over all primes with a large effect which was independent of comorbid depressive symptoms. These results are in accordance with findings of a meta-analysis in typically developing children and youth predominantly from community-based samples which found a medium to large, albeit heterogeneous, effect for positive associations of anxiety with negative interpretation bias assessed with self-report measures [71]. In the meta-analysis, age and content specificity moderated the effect sizes which were found to be larger in older individuals and when the scenario content matched the anxiety subtype. Similar to our study, comorbid psychiatric disorders or symptoms, such as depressive symptoms, did not change this finding. The present study supplements the meta-analysis by replicating the anxiety-related interpretation bias also for an implicit disorder specific task, as we tested for the pleasantness of facial expressions. The hypothesised negative correlations for the AMP total score and the explicit SPAI-C rating were only shown in the SPG. Lower pleasantness ratings of facial expressions were associated with higher explicit SP symptoms in SPAI-C. In the CG this association could not be observed; this supports the specificity of this correlation for SP and the validity of the cognitive theory of SP. The negative facial expressions may have triggered negative dysfunctional assumptions in SP and, thus, may be relevant as a risk factor for SP and for the maintenance of SP. The exact mechanisms need to be studied by longitudinal studies with a different design, as, from this study, we cannot differentiate if interpretation bias is a symptom of manifest SP or a risk factor. Furthermore, for SPG, a negative correlation for AMP total scores and the rating of general anxiety (SBB-ANZ) was observed. Lower pleasantness total scores in the AMP were associated with higher explicitly reported general anxiety. Since AMP total scores correlated with explicit SP symptoms and general anxiety, it is important to keep in mind that we cannot differentiate whether the group differences in the AMP were driven by social anxiety or by general anxiety. Future research with a larger sample size is needed to differentiate the differences more precisely. Studies on modifying interpretation bias using Cognitive Bias Modification (CBM) interventions, e.g. the Interpretation Modification Program (IMP) or CBM using positive imagery, were found to be successful in altering interpretation bias in SP and depression [72, 73]. In these studies, interpretation bias was assessed explicitly using self-report measures. To our knowledge, so far, no studies exist on the modification of interpretation bias by assessing it implicitly.

Methodically, the explicit ratings of the facial expressions did not result in the expected differences in valence and arousal between the different facial expressions, although in a validation study on these face stimuli, the Radboud Faces Data Set [55], participants recognised the displayed emotions correctly and rated their valence according to the expectation of the authors on a positive to negative continuum. In our study, participants were asked to rate valence on a SAM rating scale [52] from happy to sad. It is possible that these categories were not suitable to assess differences in valence, whereas other categories, e.g. positive to negative (as used in the validation study [55]), would have been more suitable to find differences. Second, the missing differences could also be due to the small subsample size. Thirdly, the unexpected results of arousal and valence ratings of the facial expressions could be due to less developed emotion recognition skills of youth with SP. It was found that children were less accurate in recognising the expressed emotion of the Radboud Faces [55] than adults [74]. Recognition of emotional facial expression is considered to develop with age and is also proposed to depend on the emotion expressed [75]. Since patients with SP show a tendency to avoid emotional faces when social threat is activated [76] and the onset of SP in our sample is considered early, it could be argued that this has resulted in an impairment of the ability to recognise emotional facial expression, thus, this may have led to the missing differences in arousal and valence ratings.

Several limitations should be mentioned, in particular, the rather small sample size which only allowed the detection of large group differences with a power of 80% and an alpha < 0.05. Future research will be needed with larger sample sizes to support our results. Similar to other studies on social phobia in youth, many individuals with severe, chronic SP, who were asked to participate in the study, declined participation due to their general anxiety or anxiety related to study procedures (e.g. despite detailed information, an uncertainty about what exactly they could expect possibly existed). Therefore, selection biases for participation cannot be ruled out, since factors, such as parental control or personality aspects, were not controlled for. Another limitation linked to the small sample size is that it was not possible to control for medication. Six of the participants had antidepressant medication when participating; exclusion of these participants would have led to an even smaller sample size. Antidepressant medication is unlikely to account for the effects found in this study, but may potentially have decreased the effect sizes. Another limitation concerning the sample is the possibility of other comorbid diagnoses, besides depression, influencing the results. It was not possible to control for all comorbidities due to the small sample size. By controlling for depression, we have ruled out one major influencing factor. Other comorbidities were found in in a very small number of SP participants, such as eating disorder (*n* = 2; weight > 10. BMI-percentile), somatoform disorder (*n* = 2), and ADHD (*n* = 1). As these numbers are small, and individuals with a history of an eating disorder showed a BMI-percentile > 10, it seems to be unlikely that group differences may be driven by these comorbid disorders. Given the relatively high comorbidity with other anxiety disorders [77], we cannot completely rule out that group differences may be driven by anxiety in general, and not solely by social phobia.

In addition, due to the cross-sectional design of our study, we cannot draw any causal conclusions. Therefore, for future research, longitudinal studies can be used to test whether, for example, dysfunctional assumptions are relevant as risk factors for and precede the development of clinical SP. For the implicit interpretation it would be interesting to see whether the implicit interpretation bias could also be altered by interpretation bias modification interventions or other cognitive interventions.

Taken together, implicit interpretation bias is more likely to play a role in SP, whereas implicit dysfunctional social assumptions of being rejected were not confirmed as being specific to SP, but rather for co-occurring depressive symptoms in SP. A high number of explicitly assessed symptoms of social phobia and anxiety, in general, were associated with higher implicit assumptions of being rejected and lower pleasantness ratings of facial expressions in youth with SP, when controlled for depressive symptoms. However, these results need to be interpreted with regard to the possible influence of general anxiety, since this could not be assessed separately in the present study. Other limitations of the study, e.g. the small sample size, should also be considered. Since it remains unclear whether implicit assumptions and biases are the cause, consequence or epiphenomena of SP, future research should take a closer look at causal conclusions and modification possibilities of the implicit cognitive aspects in SP.

## Supplementary Information

Below is the link to the electronic supplementary material.

Supplementary file1 (DOCX 14 KB)
