# Supplementary material for: Implicit assumptions and interpretation bias in youth with severe, chronic social phobia
Source: Eur Child Adolesc Psychiatry. 2021 Oct 1;32(3):501–12. doi: 10.1007/s00787-021-01879-3 (PMC10038968; doi:10.1007/s00787-021-01879-3)
Supplement: Supplementary file 1 — Supplementary file1 (DOCX 14 KB) [file 787_2021_1879_MOESM1_ESM.docx]

**Online Resource 1**

Versions of the Implicit Association Test, when counterbalancing the sides and order of the presented stimuli.

|  | Block 3 | | Block 5 | | |
| --- | --- | --- | --- | --- | --- |
|  | left | right | left | | right |
| Version 1 | self + rejection | others + acceptance | self + acceptance | others + rejection | |
| Version 2 | others + rejection | self + acceptance | others+ acceptance | self + rejection | |
| Version 3 | self + acceptance | others + rejection | self + rejection | others + acceptance | |
| Version 4 | others + acceptance | self + rejection | others + rejection | self + acceptance | |
